# Beliefs about marijuana use during pregnancy and breastfeeding held by residents of a Latino-majority, rural region of California

**DOI:** 10.1007/s10865-022-00299-1

**Published:** 2022-04-04

**Authors:** Linda D. Cameron, Sara E. Fleszar-Pavlović, Marisela Yepez, Rosa D. Manzo, Paul M. Brown

**Affiliations:** 1 Department of Psychological Sciences, University of California Merced, 5200 North Lake Road, Merced, CA 95343 USA; 2 Health Sciences Research Institute, University of California, Merced, CA USA; 3 Department of Public Health, University of California, Merced, CA USA

**Keywords:** Marijuana, Cannabis, Pregnancy, Breastfeeding, Latino, Risk perceptions

## Abstract

Marijuana use among pregnant and breastfeeding women is on the rise and carries risks for infant health and well-being. Decisions to use marijuana while pregnant and breastfeeding are motivated by beliefs that use poses minimal risk to infants and offers benefits to maternal users. Misperceptions and usage trend higher among disadvantaged populations. This study surveyed 401 community residents on beliefs about risks and benefits of marijuana use by pregnant and breastfeeding women. The study utilized techniques to enhance recruitment of Latino and disadvantaged residents of rural communities in California, a state where recreational marijuana use is legal. Analyses revealed substantial endorsement of beliefs about benefits and low risks of marijuana use while pregnant and breastfeeding, many of which run counter to current evidence. Misperceptions were particularly prevalent for cannabis users and male respondents. Trends in valid beliefs, while modest, were higher for Latinos and parents.

**Supplementary Information:**

The online version contains supplementary material available at 10.1007/s10865-022-00299-1.

Marijuana use among pregnant, breastfeeding, and reproductive-aged women has increased substantially in recent years (Jarlenski et al., 2017; Wang, 2016). In the United States, national rates of use by pregnant women increased from 3.4% in 2002 to 7.0% in 2017 (Volkow et al., 2019) and particularly among younger women (Oh et al., 2017). In California, marijuana use by pregnant women increased from 4.2% in 2009 to 7.1% in 2016 and particularly for women ages 18 to 24, for whom use rates rose from 9.8 to 19% over this time (Young-Wolff et al., 2017). Comparable trends of increases in marijuana use, and especially for younger women, have been identified for new mothers (Alshaarawy et al., 2020) and women of reproductive age in general (Brown et al., 2017). These increases fuel concerns about infant health consequences. The higher rates of use among young women, disadvantaged communities (Brown et al., 2017; Odom et al., 2019), and minority populations (Mark et al., 2017) highlight the potential for disparities in infant health consequences due to maternal marijuana use.

Marijuana use can be motivated by multiple psychosocial factors including peer use and efforts to cope with stress and negative affect (Fox et al., 2011; Hinnan et al., 2021; Hyman & Sinha, 2009). Beliefs about benefits and harms of marijuana use represent additional and malleable determinants of marijuana use. For example, marijuana use during pregnancy and while breastfeeding can be promoted by beliefs that it poses little or no risk to one’s infant (Odom et al., 2019) and offers benefits such as reducing nausea or depression (Changa et al., 2019). Members of low-income, low-education, and minority populations, in addition to facing adversities that can exacerbate marijuana use (Fishbein et al., 2006; Sunder et al., 2007), may be particularly likely to harbor such misperceptions due to inadequate access to health information and services (Fiscella & Sanders, 2016; Lazar & Davenport, 2018). The present study examines beliefs about the risks and benefits of marijuana use during pregnancy and breastfeeding held by residents in predominantly rural communities in California, a state that legalized recreational marijuana use in January 2018. Approximately 53.2% of the residents in this region identify as Hispanic or Latino/a/x (henceforth referred to as Latino) and another 15.4% identify as a race/ethnicity other than non-Latino White (U.S. Census Bureau, 2020). This culturally diverse region exhibits among the highest levels of income and health disparities in the U.S. (Chambers et al., 2018; Lama et al., 2018; Sodavarapu et al., 2020). Language barriers, lack of landline phones, and low access to services in these communities contribute to their under-representation in studies using traditional survey techniques. This study employed survey methods designed to reach Latino, rural, and disadvantaged residents in this region.

## Infant health harms of marijuana use while pregnant and breastfeeding

Growing evidence links marijuana use during pregnancy with adverse infant outcomes including stillbirth, miscarriage, preterm delivery, low birth weight, and need for neonatal intensive care (Coleman-Cowger et al., 2018; Gunn et al., 2016; Leemaqz et al., 2016; Petrangelo et al., 2019; Varner et al., 2014; Warshak et al., 2015). Emerging evidence also reveals its associations with deficits in attention and neurobehavioral functioning in infancy and childhood (Conner et al., 2016; Grant et al., 2018; Sharapova et al., 2018), poor intellectual performance and behavioral problems in childhood (Goldschmidt et al, 2008; Paul et al., 2021), and delinquent behaviors in adolescence (Day et al., 2011). Research on how marijuana use by breastfeeding mothers affects infants is more limited, but emerging findings have stimulated concerns that it induces health harms. Its potential to affect infant physiological function is underscored by findings that tetrahydrocannabinol (THC), the main psychoactive component, can be detected in breastmilk 6 days to six weeks after use (Baker et al., 2018; Bertrand et al., 2018; Wymore et al., 2021). A systematic review evaluating the safety of marijuana use while breastfeeding found that human and animal studies providing evidence supporting concerns about risks outnumber those finding no concerning evidence (Mourh & Rowe, 2017). In the absence of definitive evidence that it poses no health harms to infants, health organizations typically recommend a conservative approach of avoiding use while breastfeeding.

Current guidelines recommend against marijuana use while pregnant or breastfeeding (American College of Obstetricians and Gynecologists, CDC 2017; Office of the Surgeon General, USDHSS, 2019). The Academy of Breastfeeding Medicine also recommends that providers counsel pregnant and breastfeeding women and their family members on the risks and uncertainties surrounding marijuana use in breastfeeding (Reece-Stremtan & Marinelli, 2015). These guidelines tend to be vague and tentative about risks which, although in keeping with current scientific evidence, could limit their persuasiveness and especially for recipients with opposing beliefs about the safety and benefits of maternal marijuana use. Consultations with local health organizations have identified concerns about the public’s misperceptions regarding safety and the lack of evidence-based guidelines that enable pregnant and breastfeeding women to make informed decisions about marijuana use (Brown, personal communication, 2018).

### Beliefs about risks and benefits of marijuana use

Beliefs that a substance poses significant health risks are protective factors against its use (National Institute on Drug Abuse, 2020) and evidence, while limited, links low risk perceptions of cannabis use with substantially higher use rates among pregnant women (Jarlenski et al., 2017), suggesting that health communications and guidelines that enhance beliefs in the risks of marijuana use during pregnancy or while breastfeeding could discourage their use during these times. In a sample of women receiving prenatal care, declines in marijuana use were attributed to having received educational materials on cessation of marijuana use during pregnancy (Mark et al., 2016). Importantly, however, acceptance of this health information will be influenced by the existing beliefs and risk perceptions held by the recipients (Ferrer & Klein, 2015). Little is known about how much women, their romantic partners and family members, and adults in their broader social networks hold beliefs that run counter to these guidelines and so may be resistant to accepting these recommendations. Understanding the beliefs held by romantic partners, family members, and members of one’s social network is important because these people can exert considerable influence on women’s health choices in general (Holt-Lunstadt & Uchino, 2015; Perry et al., 2016), during pregnancy, and while breastfeeding (Ma et al., 2018; Masho et al., 2014; 20 Street & Lewallen, 2013). Thus, understanding the beliefs held by community members more broadly is essential for identifying common misconceptions and social groups who tend to harbor them in order to develop health communications and campaigns that promote accurate beliefs for those who influence marijuana use decisions of pregnant and breastfeeding women.

Several sources could be promoting misperceptions that marijuana use is safe and beneficial for pregnant and breastfeeding women. One potential source is social media, through which misinformation about maternal marijuana use receives increasing spread, attention, and engagement. Analyses of Twitter communications suggest an increasing engagement with misinformation about benefits of marijuana use during pregnancy and breastfeeding (Pang et al., 2021). The proliferation of online support groups advocating benefits of use while pregnant and breastfeeding (e.g., CannaMamas, GangaMamas) suggests growing popularity in beliefs that marijuana is a safe and affordable treatment for discomfort, depression, and morning sickness (for which it is particularly affordable relative to prescription medications such as *doxylamine-*pyridoxine); that it is safe because it is plant-based and natural; and that it facilitates breastfeeding. Qualitative interviews with pregnant women have documented these beliefs (Changa et al., 2019) but systematic, quantitative examinations of endorsements of these beliefs in communities remain lacking.

Another potential source of misperceptions is the legalization of marijuana for recreational and medicinal use in a growing number of states and regions. This trend could foster expectations that marijuana must be a safe substance if it is legal to use and it must be healthy if it is prescribed for medicinal purposes such as pain and depression. Among pregnant women, risk perceptions about the use of marijuana during pregnancy have decreased in recent years and these decreases might partly explain the increases in marijuana use by pregnant and breastfeeding women (Jarlenski et al., 2017; Odom et al., 2019; Oh et al., 2017). If maternal marijuana use is expected to rise in line with the increases in beliefs about its safety and benefits, then it is essential to determine which beliefs about specific risks and benefits are commonly held and which cultural and social groups are prone to misconceptions. This information can be used to identify specific beliefs to address and target audiences for health communications aimed at promoting informed decisions about using marijuana by pregnant and breastfeeding women.

### Focus on Latino, rural, and low SES communities

Whereas it is important to understand beliefs about maternal marijuana use held by adults across societies, understanding beliefs held by vulnerable populations hold particular importance given the likely health disparities if risk-elevating beliefs common within these communities are not addressed through health communications and interventions. The San Joaquin Valley is home to vulnerable, underserved communities that struggle with significant health disparities and lack of access to health care resources (Alcala et al., 2017; Larsen et al., 2017). Latinos make up the majority of residents and, more generally, represent one of the fastest-growing ethnic groups in the United States (Noe-Bustamante et al., 2020). Latinos face numerous adversities that heighten their risk of substance use (Cardoso et al., 2016; Fite et al., 2014) and have seen the most growth in marijuana use across ethnicities in the U.S. (Azofeifa et al., 2016), further indicating the need to understand and target misconceptions within these communities.

### Study aims

There is a paucity of evidence, and particularly quantitative data, on the beliefs about marijuana use while pregnant and breastfeeding held by community members, and particularly those residing in vulnerable and underserved communities. The aims of the present study were to address these research gaps by surveying community residents, with an emphasis on recruiting Latino, rural, and disadvantaged residents into the sample, to gather information about their beliefs regarding marijuana use by pregnant and breastfeeding women. The survey was designed to inform two primary research questions: (1) What beliefs about the risks and benefits of marijuana use when pregnant or breastfeeding are commonly held by adults representing these rural and vulnerable populations? And (2) How do these beliefs vary as a function of gender, ethnicity, marijuana use, and parental status? While the study aims are primarily exploratory, we made general predictions based on prior findings of social group differences in marijuana risk perceptions, substance use beliefs, or health risk perceptions more generally. We predicted that risk beliefs would be lower and benefits beliefs would be higher for participants who had ever used marijuana and who had used marijuana in the past six months relative to those who had not (Leos-Toro et al., 2020); participants who were not of Latino ethnicity (regardless of race) relative to Latino participants (Odom et al., 2019; Roppolo et al., 2019; SAMSHA 2020); male relative to female participants (Choi et al., 2018); and participants who were not parents relative to parents (Jarlenski et al., 2017).

## Method

The methods and analyses reported in this manuscript address the primary aims of the research project. The study was approved by the university’s Institutional Review Board.

### Procedure

A network of 8 *promotores de salud* (Spanish-speaking community health workers) and 20 research assistants were trained to recruit and administer the survey to residents from counties within the San Joaquin Valley, California. The promotores network enhanced opportunities to recruit “hard to reach” community members, including those who reside in isolated geographic regions. Spanish-speaking promotores and research assistants provided Spanish versions of the survey to those who preferred it. The Spanish surveys were translated and back-translated by trained translators and then validated through focus groups with Spanish-speaking promotores. Promotores and research assistants recruited residents attending local events (e.g., farmers’ markets, community fairs), community organizations (e.g., community colleges offering English language courses, farmworkers organizations), organizations serving parents (e.g., Women, Infants, and Children services, health care clinics), and home visits by promotores to community clients. Data collection took place between April 2019 and November 2019.

Promotores and research assistants distributed informational flyers inviting adults aged 18 and older to participate in a 15–20-min survey about their views on marijuana use during pregnancy and breastfeeding. Adults who expressed interest received study information and those who consented completed the survey. Participants who could not read or write in English or Spanish were offered the opportunity to have it read to them; three participants completed the survey in this manner. Upon completion, participants received debriefing information and a $20 gift card in appreciation for their time and contributions to the study.

### Measures

The survey included (in order) measures of beliefs of the potential benefits and risks of marijuana use while pregnant and breastfeeding, demographic characteristics (i.e., age, gender, race/ethnicity, education, marital and parental status, pregnancy and breastfeeding status), and marijuana use. The items assessing beliefs about benefits and risks of marijuana use while pregnant and breastfeeding were purpose-built to target beliefs identified in qualitative literature and through searches of messages on Twitter and material posted by online support groups, as well as beliefs that align with or are counter to current scientific understanding of potential benefits and harms. Tables 2–5 present the belief items; in each table, the perceived benefits (6 items) and risks (8 items) of using marijuana while pregnant are listed first, followed by perceived benefits (6 items) and risks (5 items) of marijuana use while breastfeeding. Participants rated whether they *strongly disagree, disagree*, *are* *neutral about, agree, or strongly agree* with each statement. Two additional items assessed beliefs about THC in breastmilk. One item provided the stem, “When a breastfeeding mother uses marijuana…” followed by the response options, *some THC passes from the breast milk to baby but has no effect on the baby*; *THC passes from the breastmilk to the baby and has temporary to permanent negative effects*; and *THC passes from the breastmilk to the baby and has temporary to permanent positive effects*. For the second item, the stem, “After a breastfeeding mother uses marijuana, THC will be in the breastmilk…” was followed by the response options, *0 h to 2 days*, *6 days to 2 weeks*, and *permanently*.

Finally, the survey included two marijuana use items, each with *yes* and *no* response options. First, “Have you **ever** used any marijuana products? These products may include but are not limited to joints, blunts, bongs, dabs, marijuana vapes, IQOS, marijuana edibles, and marijuana topicals or creams.” Second, “In the past 6 months, did you use any marijuana products? These products may include but are not limited to joints, blunts, bongs, dabs, marijuana vapes, IQOS, marijuana edibles, and marijuana topicals or creams.”

## Results

In total, 401 participants completed the survey. Table 1 presents demographic and personal characteristics. Participants ranged in age from 18 to 89 years, with an average age of 36 years and 80.1% aged 49 or younger. The majority identified as female, Latino, parents, and married or living with a partner. Education, an indicant of SES, was generally low with almost 50% of respondents having high school/GED or less as their highest level; an additional 9.0% did not respond to this item, which is a high rate relative to the low levels of missing values on the other demographic characteristics. About one-third of the sample completed the questionnaire in Spanish and reported speaking Spanish primarily in the home; over one-third were born outside of the United States. Overall, 6.5% of the sample were pregnant women or men with pregnant partners; 4.0% were women who reported breastfeeding or men who reported that their partners were breastfeeding, although the high rates of missing values for these items (10.7% of women and 9.1% of men) suggests uncertainty or reluctance to report this information.

**Table 1:** Demographic and marijuana use characteristics

Demographics	Total sample (*N* = *401)*	Ever used marijuana (*N* = *147)*	Used marijuana in Past 6 months *(N* = *60)*
*Age (years)*			
Mean ± SD	36.4 ± 14.3	34.1 ± 13.5	30.2 ± 11.2
*Gender*			
Female	78.8%	75.7%	78.3%
Male	19.2%	23.0%	20.0%
Other	0.5%	0.7%	0.0%
*Race/Ethnicity*			
Asian	3.5%	0%	0.0%
Black/African American	4.0%	5.4%	3.3%
Hispanic/Latino	73.6%	67.6%	75.0%
Non-Hispanic White	14.0%	20.9%	20.0%
Other	1.4%	2.1%	0.0%
Parents	69.1%	60.1%	45.0%
Married/Living with Partner	57.6%	47.3%	36.7%
*Education*			
Less than High School	24.4%	15.5%	15.0%
High School/GED	23.7%	24.3%	23.3%
Some College/Associate’s Degree	20.0%	28.4%	28.3%
Bachelor’s Degree	11.7%	14.8%	15.0%
Graduate Degree	11.5%	10.8%	10.0%
Completed Survey in Spanish	35.7%	17.6%	18.3%
Primarily Spanish Speaking Home	31.9%	16.2%	26.7%
Born Outside U.S	36.2%	15.1%	18.3%
Females who are Pregnant	5.5%	4.7%	1.7%
Females who are Breastfeeding	3.2%	3.4%	6.7%
Males with Pregnant Partner	1.0%	1.4%	1.7%
Males with Breastfeeding Partner	0.5%	0.7%	0.0%

For demographic variables, the proportions of missing values ranged from 0% to 2.5% with the exceptions of education (8.7%), females who are breastfeeding (10.7%), and males with partners who are breastfeeding (9.1%). For marijuana use variables, the proportions of missing values were 1.2%

Overall, 36.1% had ever used marijuana and 15.0% had used it in the past six months; of those who had ever used marijuana, 40.5% had used it in the past six months. Those who had ever (versus never) used marijuana were younger (*M* = 34.09 versus *M* = 37.90, *t*(387) = 2.57, *p* = 0.010) and less likely to have completed high school (67.1% vs. 83.3%, χ^2^ [1, *N* = 363] = 11.50, *p* = 0.001), be parents (59.9% vs. 76.3%, χ^2^ [1, *N* = 392] = 11.89, *p* = 0.001), and be married or living with a partner (47.6% vs. 66.5%, χ^2^ [1, *N* = 389] = 13.56, *p* < 0.001); they were also more likely to be born in the U.S. (84.5% vs. 49.6%, χ^2^ [1, *N* = 394] = 48.03, *p* < 0.001). These groups did not differ in race/ethnicity and gender. Those who had (versus had not) used marijuana in the past six months also were younger (*M* = 30.22 versus *M* = 34.98, *t*(208) = 2.39, *p* = 0.020) and less likely to be parents (45.8% vs. 70.5%, χ^2^ [1, *N* = 215] = 11.34, *p* = 0.001) and married or living with a partner (37.3% vs. 56.2%, χ^2s^ [1, *N* = 212] = 6.10, *p* = 0.014). These groups did not differ in race/ethnicity, gender, education, and birth in versus outside of the U.S.

### Perceived benefits and risks of marijuana use while pregnant and breastfeeding

Table 2 presents rates of agreement with statements about benefits and risks of marijuana use while pregnant or breastfeeding for the total sample. A slight majority (51.0%) were either neutral about or agreed with the statement that using marijuana while pregnant helps to reduce pain and discomfort whereas 49% disagreed with this statement. In contrast, a strong majority (64.1–74.2%) of participants disagreed that marijuana use during pregnancy helps to reduce depression, has no lasting harms for the baby, is safe because it is plant-based and natural, and helps to reduce morning sickness and nausea. Over 60% of participants agreed that use during pregnancy poses risks to the baby including attention and learning difficulties, lowered IQ, THC addiction, behavioral problems, brain damage, preterm birth, low birth weight, and pregnancy complications. Proportions who were neutral or disagreed with these statements ranged from 29.0% for risk of behavioral problems to 39.0% for risks of preterm birth and low birth weight.

**Table 2:** Agreement with perceived benefits and harms of marijuana using while pregnant and breastfeeding

	*Total Sample (N* = *401)*		
	Strongly Disagree/Disagree (%)	Neutral (%)	Agree/Strongly Agree (%)
*Using marijuana while pregnant…*			
Helps to reduce pain and discomfort	49.0	21.2	29.8
Helps to reduce depression	64.1	19.3	16.5
Has no lasting harms for baby	74.2	9.7	16.1
Is safe because marijuana is plant-based, natural	68.1	19.2	12.7
Helps reduce morning sickness, nausea	71.5	14.9	13.6
Average % for perceived benefits while pregnant	65.4	16.9	17.7
Makes it hard for child to pay attention, learn	17.9	16.7	65.4
Lowers child’s IQ	18.2	17.9	63.9
Leads to baby being addicted to THC	18.8	14.4	66.8
Increases risk of behavioral problems	15.9	13.1	71.0
Increases risk of damage to baby’s brain	14.7	12.6	72.8
Increases risk of preterm birth	24.1	14.9	61.0
Increases risk of low birth weight	23.3	15.7	61.0
Increases risk of pregnancy complications	18.9	14.8	66.3
Average % for perceived harms while pregnant	19.0	15.0	66.0
*Using marijuana while breastfeeding…*			
Helps to reduce pain and discomfort	46.1	17.8	36.1
Helps to reduce depression	55.8	21.2	23.0
Has no lasting harms for baby	70.9	16.8	12.3
Is safe because marijuana is plant-based, natural	72.8	18.0	9.3
Helps to increase mother’s milk supply	71.5	19.7	8.7
Helps calm the baby	75.3	16.6	8.1
Average % for perceived benefits while breastfeeding	65.4	18.4	16.3
Makes it hard for child to pay attention, learn	23.5	15.2	61.2
Lowers the child’s IQ	21.2	18.1	60.6
Leads to baby being addicted to THC	20.6	16.0	63.4
Increases risk of behavioral problems	19.9	12.7	67.4
Increases risk of damage to baby’s brain	18.4	13.5	68.1
Average % for perceived harms while breastfeeding	20.7	15.1	64.1

Comparable patterns of agreement emerged for beliefs about use while breastfeeding. A slight majority (53.9%) were neutral about or agreed that it helps to reduce pain and discomfort whereas a majority disagreed with the other five statements about benefits. Proportions of participants who were neutral or agreed with these five statements ranged from 24.7% for use helps calm the baby to 44.2% for use helps reduce depression. Over 60% agreed that use while breastfeeding poses risks to the baby including attention and learning difficulties, lowered IQ, THC addiction, behavioral problems, and brain damage. Proportions of participants who were neutral or disagreed with these statements ranged from 29.0% for risk of attention and learning difficulties to 31.9% for risk of brain damage.

In response to items assessing beliefs about the presence and effects of THC in breastmilk, 65.6% of participants believed that THC passes to the baby and has negative effects on the baby (beliefs that are in line with scientific knowledge) whereas 21.0% believed it passes to the baby and has positive effects on the baby and 13.4% believed that none to some THC passes to the baby and has no effect on the baby. Only 20% of participants gave the most scientifically accurate response that THC lasts in breastmilk for 6 days to 2 weeks; 42% believed it lasts 0 h to 2 days and 37.3% believed it stayed in breastmilk permanently.

### Differences in beliefs by marijuana use, Latino ethnicity, gender, and parental status

We used *χ*^2^ tests to determine differences in agreement (strongly disagree/disagree, neutral, agree/strongly agree) with potential risks and benefits of marijuana use during pregnancy and breastfeeding as a function of marijuana use, Latino ethnicity, gender, and parental status. Table 3 presents the proportions of agreement with benefits and risk statements by participants who had ever versus never used marijuana. As predicted, those who had ever (versus never) used marijuana were significantly more likely to be neutral about or agree with all statements about potential benefits of marijuana use while pregnant or breastfeeding, and to be neutral about or disagree with all statements about potential health risks of marijuana use while pregnant or breastfeeding. Analyses of differences between participants who had used marijuana in the past 6 months and those who had not revealed similar patterns of group differences. Those who had used marijuana in the past 6 months reported higher agreement with all benefits statements (with one exception: use increases a mother’s milk supply) and lower agreement with all risk statements for marijuana use in pregnancy and while breastfeeding (see Table S1 in supplementary materials).

**Table 3:** Agreement with perceived benefits and harms of marijuana use while pregnant and breastfeeding by participants who had ever versus never used marijuana

	*Ever Used Marijuana (N* = *148)*			*Never Used Marijuana (N* = *245)*			
	Strongly Disagree/Disagree (%)	Neutral (%)	Agree/Strongly Agree (%)	Strongly Disagree/Disagree (%)	Neutral (%)	Agree/Strongly Agree (%)	*χ*^2^
*Using marijuana while pregnant…*							
Helps to reduce pain and discomfort	38.4	19.9	41.8	55.1	22.2	22.6	16.71^***^
Helps to reduce depression	53.7	23.1	23.1	70.4	17.3	12.3	12.05^**^
Has no lasting harms for baby	68.8	16.7	14.6	77.5	5.7	16.8	12.25^**^
Is safe because marijuana is plant-based, natural	59.3	24.1	16.6	73.3	16.6	10.1	8.32^*^
Helps reduce morning sickness, nausea	57.1	19.7	23.1	80.2	12.1	7.7	26.67^***^
Makes it hard for child to pay attention, learn	20.7	24.1	55.2	15.7	12.5	71.8	12.51^**^
Lowers child’s IQ	24.0	22.6	53.4	14.2	15.4	70.4	11.74^**^
Leads to baby being addicted to THC	31.0	20.0	49.0	10.8	11.2	78.0	36.38^***^
Increases risk of behavioral problems	20.7	23.4	55.9	12.4	7.0	80.6	30.35^***^
Increases risk of damage to baby’s brain	18.8	19.4	61.8	11.6	8.7	79.8	15.49^***^
Increases risk of preterm birth	24.0	24.7	51.4	23.6	9.3	67.1	17.96^***^
Increases risk of low birth weight	26.0	24.0	50.0	21.5	11.0	67.5	15.17^***^
Increases risk of pregnancy complications	18.1	25.0	56.9	18.8	9.0	72.2	18.83^***^
*Using marijuana while breastfeeding…*							
Helps to reduce pain and discomfort	32.4	17.9	49.7	54.2	17.9	27.9	21.13^***^
Helps to reduce depression	40.0	27.6	32.4	65.0	17.7	17.3	23.43^***^
Has no lasting harms for baby	57.4	25.5	17.0	79.1	11.7	9.1	20.08^***^
Is safe because marijuana is plant-based, natural	61.1	26.4	12.5	79.8	13.2	7.0	15.92^***^
Helps to increase mother’s milk supply	65.7	26.6	7.7	75.0	16.0	9.0	6.35^*^
Helps calm the baby	62.5	28.5	9.0	83.2	9.7	7.1	24.45^***^
Makes it hard for child to pay attention, learn	27.5	20.4	52.1	20.7	12.4	66.9	8.74^*^
Lowers the child’s IQ	23.9	26.1	50.0	18.7	13.7	67.6	13.23^***^
Leads to baby being addicted to THC	31.0	22.5	46.5	13.6	12.3	74.1	30.04^***^
Increases risk of behavioral problems	25.4	22.5	52.1	15.7	7.0	77.3	29.53^***^
Increases risk of damage to baby’s brain	24.6	18.3	57.0	14.1	10.8	75.1	13.50^***^

*Note.* *p < .050, **p < .010, ***p < .001. Of the total sample, five participants did not report their marijuana use and so their responses were not included in these analyses

Latino and non-Latino participants were generally comparable in their agreement about benefits and risks of marijuana use while pregnant and breastfeeding, with the following exceptions in which, as predicted, Latino participants reported relatively more health-cautious beliefs (see Table 4). Fewer Latino than non-Latino respondents endorsed the beliefs that marijuana use during pregnancy reduces pain and discomfort and that it reduces morning sickness and nausea, and more Latino than non-Latino respondents agreed that use could lower a child’s IQ. For statements about marijuana use while breastfeeding, fewer Latino than non-Latino respondents were neutral about or agreed that it helps calm the baby and more Latino than non-Latino respondents believed that use increases the risk of attention and learning difficulties.

**Table 4:** Agreement of Non-Latino and Latino Respondents with Perceived Benefits and Harms of Marijuana Use While Pregnant and Breastfeeding

	Non-Latino Respondents (N = *101)*			*Latino Respondents (N* = *295)*			
	Strongly Disagree/Disagree (%)	Neutral (%)	Agree/Strongly Agree (%)	Strongly Disagree/Disagree (%)	Neutral (%)	Agree/Strongly Agree (%)	*χ*^2^
*Using marijuana while pregnant…*							
Helps to reduce pain and discomfort	37.1	24.7	38.1	52.1	19.9	28.0	6.61^*^
Helps to reduce depression	60.0	21.1	19.0	65.3	18.6	16.1	0.87
Has no lasting harms for baby	79.8	10.6	9.6	71.8	9.5	18.7	4.25
Is safe because marijuana is plant-based, natural	69.5	15.8	14.7	67.2	20.2	12.5	1.04
Helps reduce morning sickness, nausea	57.7	25.8	16.5	75.6	11.5	12.9	13.72^***^
Makes it hard for child to pay attention, learn	18.8	20.8	60.4	17.8	15.0	67.2	2.05
Lowers child’s IQ	22.9	24.0	53.1	16.7	15.7	67.6	6.64^*^
Leads to baby being addicted to THC	22.8	18.5	58.7	17.5	13.0	69.5	3.71
Increases risk of behavioral problems	18.5	14.1	67.4	15.0	12.2	72.7	0.99
Increases risk of damage to baby’s brain	17.4	13.0	69.6	13.7	11.6	74.7	1.03
Increases risk of preterm birth	18.9	16.8	64.2	25.4	13.9	60.6	1.82
Increases risk of low birth weight	18.5	17.7	63.5	24.1	15.0	60.8	1.34
Increases risk of pregnancy complications	16.8	3.2	68.4	18.7	14.8	66.5	0.17
*Using marijuana while breastfeeding…*							
Helps to reduce pain and discomfort	37.2	17.0	45.7	48.2	18.1	33.7	4.71
Helps to reduce depression	58.1	14.0	28.0	54.2	24.1	21.7	4.72
Has no lasting harms for baby	70.3	15.4	14.3	71.0	16.9	12.1	0.35
Is safe because marijuana is plant-based, natural	64.9	22.3	12.8	75.3	16.6	8.1	3.97
Helps to increase mother’s milk supply	71.3	21.3	7.4	71.1	19.4	9.5	0.46
Helps calm the baby	63.8	27.7	8.5	78.6	13.2	8.2	10.77^**^
Makes it hard for child to pay attention, learn	26.1	22.8	51.1	21.9	12.7	65.4	7.83^*^
Lowers the child’s IQ	25.0	19.6	55.4	19.9	17.4	62.8	1.67
Leads to baby being addicted to THC	23.9	19.6	56.5	20.1	14.8	65.1	2.30
Increases risk of behavioral problems	21.7	13.0	65.2	19.8	12.4	67.8	0.28
Increases risk of damage to baby’s brain	22.8	13.0	64.1	16.7	13.5	69.9	1.79

*p < .050, **p < .010, ***p < .001. Of the total sample, 15 participants did not identify their ethnicity and so their responses were not included in these analyses

In terms of gender differences, predictions that benefits beliefs would be higher and risk beliefs would be lower for male than female respondents were supported for 10 of the 24 beliefs (see Table 5). More males than females agreed that marijuana use during pregnancy reduces pain and discomfort whereas more females than males agreed that marijuana use during pregnancy increases the risks of preterm birth and low birth weight. With respect to marijuana use during breastfeeding, more males than females agreed that it helps to reduce pain and depression, poses no lasting harms to the baby, is safe because marijuana is plant-based and natural, and increases a mother’s milk supply. In contrast, more females than males indicated agreement that use during breastfeeding can lead to infant THC addiction and increase the risk of behavior problems.

**Table 5:** Agreement of Female and Male Respondents with Perceived Benefits and Harms of Marijuana Use While Pregnant and Breastfeeding

	Female Respondents (N = 316)			Male Respondents (N = 77)			
	Strongly Disagree/Disagree (%)	Neutral (%)	Agree/Strongly Agree (%)	Strongly Disagree/Disagree (%)	Neutral (%)	Agree/Strongly Agree (%)	*χ*^2^
*Using marijuana while pregnant…*							
Helps to reduce pain and discomfort	54.5	19.4	26.1	26.3	27.6	46.1	19.97^***^
Helps to reduce depression	67.1	18.4	14.5	54.5	22.1	23.4	4.92
Has no lasting harms for baby	74.4	9.0	16.7	74.0	12.3	13.7	1.02
Is safe because marijuana is plant-based, natural	70.5	18.3	11.2	61.0	19.5	19.5	4.14
Helps reduce morning sickness, nausea	73.6	13.7	12.7	64.9	19.5	15.6	2.41
Makes it hard for child to pay attention, learn	17.3	14.7	68.1	19.5	22.1	58.4	3.12
Lowers child’s IQ	17.6	16.0	66.5	19.5	24.7	55.8	3.85
Leads to baby being addicted to THC	17.0	13.8	69.1	27.4	16.4	56.2	5.11
Increases risk of behavioral problems	16.1	12.9	71.1	14.7	13.3	72.0	0.09
Increases risk of damage to baby’s brain	15.2	11.0	73.8	12.0	17.3	70.7	2.48
Increases risk of preterm birth	23.3	11.8	64.9	25.0	26.3	48.7	11.43^**^
Increases risk of low birth weight	23.0	11.2	65.8	25.0	31.6	43.4	21.87^***^
Increases risk of pregnancy complications	17.6	14.7	67.6	21.6	13.5	64.9	0.65
*Using marijuana while breastfeeding…*							
Helps to reduce pain and discomfort	50.5	16.5	33.0	28.9	22.4	48.7	11.46^**^
Helps to reduce depression	59.4	19.0	21.6	42.1	28.9	28.9	7.53^*^
Has no lasting harms for baby	74.2	14.8	11.1	58.9	23.3	17.8	6.65^*^
Is safe because marijuana is plant-based, natural	75.3	16.9	7.8	63.2	21.1	15.8	6.00^*^
Helps to increase mother’s milk supply	73.5	16.5	10.0	67.1	30.3	2.6	10.25^**^
Helps calm the baby	77.1	14.7	8.2	70.3	23.0	6.8	3.02
Makes it hard for child to pay attention, learn	22.7	14.9	62.3	28.0	14.7	57.3	0.96
Lowers the child’s IQ	20.6	17.3	62.1	25.3	20.0	54.7	1.42
Leads to baby being addicted to THC	18.1	13.9	68.0	32.4	23.0	44.6	14.12^***^
Increases risk of behavioral problems	18.2	11.1	70.7	28.0	17.3	54.7	7.06^*^
Increases risk of damage to baby’s brain	17.0	12.8	70.2	25.0	13.2	61.8	2.69

*p < .050, **p < .010, ***p < .001. Of the total sample, eight participants did not identify their gender or identified as a gender other than male or female and so their responses were not included in these analyses

Parents were generally comparable to non-parents in their beliefs about the benefits and risks of marijuana use, differing only on four beliefs about risks of use while pregnant and two beliefs about effects of use while breastfeeding. Parents (versus non-parents) reported higher agreement that marijuana use during pregnancy poses risks of attention and learning difficulties (68.7% versus 58.1%; *χ*^2^ [2, *N* = 395] = 7.32, *p* < 0.05); lowered IQ (67.9% versus 55.1%; *χ*^2^ [2, *N* = 395] = 9.33, *p* < 0.010); preterm birth (63.7% versus 55.1%; *χ*^2^ [2, *N* = 395] = 16.25, *p* < 0.001), and low birth weight (64.4% versus 53.4%; *χ*^2^ [2, *N* = 395] = 11.88, *p* < 0.001). Parents also reported relatively higher agreement that marijuana use during breastfeeding could increase a mother’s milk supply (10.7% versus 4.3%, *χ*^2^ [2, *N* = 395] = 8.43, *p* < 0.050) but lower the child’s IQ (62.9% versus 55.2%; *χ*^2^ [2, *N* = 395] = 6.95, *p* < 0.050). Proportions for agreement with all belief statements by parental status are available in supplementary materials (Table S2).

Finally, we conducted *χ*^2^ tests of responses to items assessing beliefs about the presence and effects of THC in breastmilk. Responses to these items did not vary by marijuana user status, Latino ethnicity, gender, parental status, or education level (0.23 < *χ*^2^ < 5.77, all *p*’s > 0.560).

## Discussion

The present study revealed distinctive patterns of beliefs about marijuana use while pregnant and breastfeeding in a sample of residents in primarily rural, Latino-majority, and disadvantaged communities in California, a state that has legalized the use of marijuana for recreational and medicinal purposes. Taken together, the levels of endorsement of beliefs about benefits of use and lack of endorsement of beliefs about risks to infant health and well-being highlight the need for public health education about risks of maternal marijuana use in these communities and identify specific beliefs and community groups to prioritize in these educational efforts.

Overall, the most common beliefs regarding marijuana use both during pregnancy and while breastfeeding are that it helps to reduce pain and depression. These beliefs are not supported by empirical research. In contrast with modest support that cannabis can reduce pain for people with chronic pain conditions (Andreae et al., 2015), evidence that it reduces pain sensitivity is inconsistent (Allan et al., 2018; Mun et al., 2020), and research on the effects of marijuana on pain during pregnancy and while breastfeeding remains lacking. For depression, the evidence base suggests that cannabis can worsen rather than improve depression (Chadwick et al., 2020; Whiting et al., 2015) and, to our knowledge, no studies have tested its impact on depressive symptoms for pregnant and breastfeeding women.

Almost 30% of participants reported neutral or positive beliefs that marijuana reduces nausea during pregnancy, another belief that is not empirically supported. Although some evidence suggests that cannabis can aid in pain relief and nausea in cancer patients (Whiting et al., 2015), it is also positively linked with episodes of nausea and vomiting in cancer patients (Whiting et al., 2015) and the general public (Bollom et al., 2018). Despite perceptions that cannabis reduces nausea during pregnancy (Jarlenski et al., 2018; Westfall et al., 2006) and prevalent advice from cannabis dispensaries to use cannabis to alleviate pregnancy-related nausea (e.g., 70% of dispensaries in Colorado do so; Dickson, 2018), evidence remains lacking.

In addition to the substantial endorsement of beliefs that use provides symptom relief for maternal users, up to 25% of participants were neutral about or agreed that it is safe because it is plant-based and natural and that it poses no lasting harms to the infant. Beliefs that use while breastfeeding helps calm the baby received the least endorsement, suggesting that it might be given lower priority in health communications and guidelines. Overall, these survey results are consistent with qualitative findings of benefit beliefs held by pregnant women (Changa et al., 2019) and demonstrate that they are shared by community members more generally.

The majority of respondents were cognizant of the risks of maternal marijuana use for fetuses and infants, yet sizeable proportions (between 28% and 40%) disagreed with or were neutral about the validity of these risks. For beliefs about marijuana use during pregnancy, the evidenced-based risks of preterm birth and low birth weight received the lowest levels of agreement and thus warrant high prioritization in health communications, educational materials, and guidelines. For beliefs about marijuana use while breastfeeding, attention and learning problems along with detrimental effects on intellectual capabilities received the lowest endorsement. Given the growing evidence for these cognitive consequences and their adverse impacts throughout the lifespan, public health communications should aim to increase awareness of their potential while reporting the current status of empirical support.

The findings suggest that ever users, and not just recent users, are important targets for health information about maternal marijuana use. Specifically, prior cannabis users (relative to non-users) reported more supportive beliefs about its benefits and more lenient beliefs about its risks. These patterns were found not only for recent marijuana users but also for those who had ever used marijuana. These findings suggest that prenatal and postnatal providers could ask about the history of *any* prior marijuana use to become more informed about one maternal feature that could increase the propensity to be open to using marijuana.

Similarly, the findings suggest that males are particularly likely to benefit from education about the risks of maternal marijuana use. Important targets include the risks of preterm birth and low birth weight, the lack of evidence about its benefits in reducing pain, the potential harms for breastfeeding babies despite marijuana being plant-based, its lack of impact on milk supply, and the potential for infant THC addiction.

Latino and non-Latino respondents differed on only four beliefs about maternal marijuana use, although these differences were in the predicted direction and consistent with evidence that Latinos tend to hold more cautious beliefs about marijuana harms and benefits relative to other ethnic groups (Odom et al., 2017; Roppolo et al., 2019; SAMSHA, 2020). Latino respondents held more cautious beliefs about its benefits in reducing pain and nausea during pregnancy and calming the baby when breastfeeding, and the risks of child attention and learning deficits with use while breastfeeding. The findings provide no support that, in rural, Latino-majority, disadvantaged communities, Latino residents are less well-informed than non-Latino residents on these health issues. Having Spanish-fluent research assistants and promotores, who are highly trusted in these communities, recruit residents and offer Spanish versions of the survey materials likely protected against ethnic group differences arising from poor comprehension or survey engagement.

Few differences between parents and non-parents emerged although they were consistent with predictions that parents would have relatively more cautious beliefs about the benefits and risks of maternal marijuana use. This pattern is consistent with findings from a prior study that parents (relative to non-parents) perceived marijuana use by pregnant women as more risky and less beneficial (Jarlenski et al., 2017). Importantly, however, this prior study included a generic measure of risk and thus lacked the specificity afforded by the items in the present study. The present findings thus provide a more nuanced and detailed framework of specific risk and benefits beliefs, highlighting the few that are likely to be strong contributors to parental status differences in general risk perceptions of maternal marijuana use.

Key strengths and limitations of the study warrant attention in contextualizing the findings and highlighting future research directions. The study successfully recruited a large community sample with strong representation of Latino, female, and Spanish-preference residents of disadvantaged, rural communities and thus contributes to efforts to build the scientific evidence base with data from these and other under-represented communities (Schuz & Webb Hopper, 2020). However, the sample included a limited number of male, pregnant, and breastfeeding residents. Further, the relatively high rates of missing values (9.0 to 10.7% of the sample) for reports of education, pregnancy status, and breastfeeding status contribute modest uncertainty about these sample characteristics. Social desirability concerns could inhibit motivations to report these characteristics. In addition, information about social ties with pregnant and breastfeeding women other than partner status was not collected. Future studies can extend the current research by using a sample with a larger number of male residents as well as measures that minimize social desirability and that clearly identify pregnant and breastfeeding women and those who are partners, family members, health workers, and influencers in their social networks. In addition, research utilizing a nationally representative sample is needed to provide a common benchmark for interpreting and contextualizing regional and social group patterns of beliefs. There is also a need for more research on how partners, family members, friends, and members of the broader community influence decisions to use marijuana while pregnant and breastfeeding. This research can inform the development of health communications and tools that enable influential people in social networks and communities to engage with women to promote and support their informed decisions on marijuana use. Future research could include measures of health care advice and education about marijuana use during pregnancy and breastfeeding to discern how it is associated with beliefs about its harms and benefits.

As the U.S. and other nations transition to legalizing recreational marijuana, there is a growing need for science-based guidelines for counseling and public education efforts to increase awareness about the health effects of marijuana use. Pregnant and breastfeeding women, along with their partners, families, and friends, highly desire healthy infants and want to learn about potential risks and ways to reduce them (Changa et al., 2019; Dakkak et al., 2018). These findings provide insights into beliefs about maternal marijuana use held by members of the diverse, rural regions in California. These belief frameworks can inform the development of health communications and guidelines about risks of use during pregnancy and breastfeeding so that they target common misperceptions and are tailored to demographic and social groups.

## Supplementary Information

Below is the link to the electronic supplementary material.

Supplementary file1 (DOCX 25 kb)
