# Supplementary material for: Beliefs about marijuana use during pregnancy and breastfeeding held by residents of a Latino-majority, rural region of California
Source: J Behav Med. 2022 Apr 4;45(4):544–57. doi: 10.1007/s10865-022-00299-1 (PMC9304043; doi:10.1007/s10865-022-00299-1)
Supplement: Supplementary file 1 — Supplementary file1 (DOCX 25 kb) [file 10865_2022_299_MOESM1_ESM.docx]

Supplementary Materials

Table S1

*Agreement with Perceived Benefits and Harms of Marijuana Use While Pregnant and Breastfeeding by Participants Who Used and Had Not Used Marijuana in the Past Six Months*

|  | *Past 6-Months Users (N=60)* | | | *Non-Users in Past 6-Months (N=156)* | | |  |
| --- | --- | --- | --- | --- | --- | --- | --- |
| **Using marijuana while pregnant…** | Strongly Disagree/ Disagree | Neutral | Agree/ Strongly Agree | Strongly Disagree/ Disagree | Neutral | Agree/ Strongly Agree | ꭓ^2^ |
| Helps to reduce pain and discomfort | 25.0% | 20.0% | 55.0% | 50.7% | 18.7% | 30.7% | 13.30*** |
| Helps to reduce depression | 35.0% | 30.0% | 35.0% | 65.6% | 17.5% | 16.9% | 16.73*** |
| Has no lasting harms for baby | 55.2% | 22.4% | 22.4% | 76.3% | 8.6% | 15.1% | 10.48** |
| Is safe because marijuana is plant-based, natural | 44.1% | 32.2% | 23.7% | 66.2% | 20.1% | 13.6% | 8.78* |
| Helps reduce morning sickness, nausea | 43.3% | 15.0% | 41.7% | 70.3% | 18.1% | 11.6% | 24.80*** |
| Makes it hard for child to pay attention, learn | 32.8% | 31.0% | 36.2% | 18.1% | 16.1% | 65.8% | 15.18** |
| Lowers child’s IQ | 40.7% | 20.3% | 39.0% | 16.8% | 21.9% | 61.3% | 14.36** |
| Leads to baby being addicted to THC | 50.8% | 20.3% | 28.8% | 19.1% | 16.4% | 64.5% | 25.62*** |
| Increases risk of behavioral problems | 39.0% | 25.4% | 35.6% | 15.0% | 14.4% | 70.6% | 22.80*** |
| Increases risk of damage to baby’s brain | 34.5% | 20.7% | 44.8% | 13.7% | 13.1% | 73.2% | 16.11*** |
| Increases risk of preterm birth | 39.0% | 30.5% | 30.5% | 20.8% | 14.9% | 64.3% | 19.71*** |
| Increases risk of low birth weight | 42.4% | 27.1% | 30.5% | 18.3% | 18.3% | 63.4% | 19.96*** |
| Increases risk of pregnancy complications | 37.9% | 31.0% | 31.0% | 15.1% | 15.8% | 69.1% | 25.44*** |
| **Using marijuana while breastfeeding…** |  |  |  |  |  |  |  |
| Helps to reduce pain and discomfort | 21.7% | 16.7% | 61.7% | 44.1% | 17.8% | 38.2% | 11.06** |
| Helps to reduce depression | 28.8% | 28.8% | 42.4% | 51.9% | 22.1% | 26.0% | 9.58** |
| Has no lasting harms for baby | 47.4% | 26.3% | 26.3% | 69.1% | 18.8% | 12.1% | 9.43** |
| Is safe because marijuana is plant-based, natural | 44.8% | 32.8% | 22.4% | 70.6% | 19.6% | 9.8% | 12.57** |
| Helps to increase mother’s milk supply | 58.6% | 31.0% | 10.3% | 68.0% | 20.3% | 11.8% | 2.74 |
| Helps calm the baby | 50.8% | 37.3% | 11.9% | 71.2% | 18.3% | 10.5% | 9.29* |
| Makes it hard for child to pay attention, learn | 34.5% | 24.1% | 41.4% | 25.8% | 15.9% | 58.3% | 4.91 |
| Lowers the child’s IQ | 37.9% | 27.6% | 34.5% | 19.9% | 19.2% | 60.9% | 12.33** |
| Leads to baby being addicted to THC | 49.1% | 22.8% | 28.1% | 20.4% | 20.4% | 59.2% | 20.16*** |
| Increases risk of behavioral problems | 35.1% | 26.3% | 38.6% | 21.6% | 13.7% | 64.7% | 11.76** |
| Increases risk of damage to baby’s brain | 35.1% | 21.1% | 43.9% | 19.1% | 13.8% | 67.1% | 9.59** |

*Note.* *p < .050, **p < .010, ***p < .001. Of the total sample, five participants did not identify their marijuana use and so their responses were not included in these analyses.

Table S2

*Agreement with Perceived Benefits and Harms of Marijuana Use While Pregnant and Breastfeeding by Participants Who Were Parents and Non-Parents*

|  | *Not Parents (N = 118)* | | | *Parents (N = 277)* | | |  |
| --- | --- | --- | --- | --- | --- | --- | --- |
| **Using marijuana while pregnant…** | Strongly Disagree/ Disagree | Neutral | Agree/ Strongly Agree | Strongly Disagree/ Disagree | Neutral | Agree/ Strongly Agree | ꭓ^2^ |
| Helps to reduce pain and discomfort | 44.9% | 21.2% | 33.9% | 51.5% | 20.7% | 27.8% | 1.76 |
| Helps to reduce depression | 56.0% | 23.3% | 20.7% | 68.2% | 17.2% | 14.6% | 5.33 |
| Has no lasting harms for baby | 73.7% | 11.4% | 14.9% | 75.1% | 8.8% | 16.1% | 0.67 |
| Is safe because marijuana is plant-based, natural | 62.7% | 25.4% | 11.9% | 71.2% | 16.1% | 12.8% | 4.75 |
| Helps reduce morning sickness, nausea | 66.7% | 17.1% | 16.2% | 73.9% | 13.8% | 12.3% | 2.15 |
| Makes it hard for child to pay attention, learn | 17.9% | 23.9% | 58.1% | 18.2% | 13.1% | 68.7% | 7.32^*^ |
| Lowers child’s IQ | 18.6% | 26.3% | 55.1% | 18.2% | 13.9% | 67.9% | 9.33^**^ |
| Leads to baby being addicted to THC | 23.9% | 16.2% | 59.8% | 16.7% | 13.4% | 69.9% | 3.94 |
| Increases risk of behavioral problems | 15.4% | 17.9% | 66.7% | 16.3% | 10.7% | 73.0% | 3.78 |
| Increases risk of damage to baby’s brain | 16.2% | 12.8% | 70.9% | 14.1% | 12.3% | 73.6% | 0.35 |
| Increases risk of preterm birth | 19.5% | 25.4% | 55.1% | 26.4% | 9.9% | 63.7% | 16.25^***^ |
| Increases risk of low birth weight | 21.6% | 25.0% | 53.4% | 24.4% | 11.3% | 64.4% | 11.88^**^ |
| Increases risk of pregnancy complications | 14.8% | 20.0% | 65.2% | 20.9% | 12.1% | 67.0% | 5.13 |
| **Using marijuana while breastfeeding…** |  |  |  |  |  |  |  |
| Helps to reduce pain and discomfort | 40.2% | 16.2% | 43.6% | 49.1% | 18.6% | 32.3% | 4.53 |
| Helps to reduce depression | 47.9% | 26.5% | 25.6% | 59.4% | 18.8% | 21.8% | 4.76 |
| Has no lasting harms for baby | 68.4% | 19.3% | 12.3% | 72.4% | 15.6% | 12.1% | 0.84 |
| Is safe because marijuana is plant-based, natural | 67.2% | 22.4% | 10.3% | 75.3% | 15.9% | 8.9% | 2.87 |
| Helps to increase mother’s milk supply | 69.2% | 26.5% | 4.3% | 73.0% | 16.3% | 10.7% | 8.43^*^ |
| Helps calm the baby | 71.6% | 21.6% | 6.9% | 77.2% | 14.2% | 8.6% | 3.27 |
| Makes it hard for child to pay attention, learn | 20.7% | 21.6% | 57.8% | 25.0% | 12.3% | 62.7% | 5.53 |
| Lowers the child’s IQ | 19.0% | 25.9% | 55.2% | 22.5% | 14.6% | 62.9% | 6.95^*^ |
| Leads to baby being addicted to THC | 22.4% | 19.0% | 58.6% | 20.1% | 14.5% | 65.4% | 1.83 |
| Increases risk of behavioral problems | 17.2% | 17.2% | 65.5% | 21.3% | 10.4% | 68.3% | 3.74 |
| Increases risk of damage to baby’s brain | 15.5% | 15.5% | 69.0% | 19.5% | 12.4% | 68.2% | 1.31 |

*Note.* *p < .050, **p < .010, ***p < .001. Of the total sample, six participants did not identify their parental status and so their responses were not included in these analyses.
